# Effects of Ground Floor Type on Selected Health-Parameters and Weight of Rabbits Reared in Group Pens

**DOI:** 10.3390/ani9050216

**Published:** 2019-05-04

**Authors:** Ines Windschnurer, Susanne Waiblinger, Stefan Hanslik, Andrea Klang, Fehim Smajlhodzic, Michael Löwenstein, Knut Niebuhr

**Affiliations:** 1 Institute of Animal Welfare Science, Department for Farm Animals and Veterinary Public Health, University of Veterinary Medicine, Vienna, Veterinärplatz 1, 1210 Vienna, Austria; Susanne.Waiblinger@vetmeduni.ac.at (S.W.); stefan@goprimal.eu (S.H.); Fehim.Smajlhodzic@gmail.com (F.S.); Knut.Niebuhr@vetmeduni.ac.at (K.N.); 2 Institute of Pathology, Department for Pathobiology, University of Veterinary Medicine, Vienna, Veterinärplatz 1, 1210 Vienna, Austria; Andrea.Klang@vetmeduni.ac.at; 3 Institute of Parasitology, Department for Pathobiology, University of Veterinary Medicine, Vienna, Veterinärplatz 1, 1210 Vienna, Austria; Michael.Loewenstein@vetmeduni.ac.at

**Keywords:** growing rabbits, pen housing, floor, slatted plastic floor, straw litter, soiling, coccidia, causes of loss, mortality, weight

## Abstract

**Simple Summary:**

Cage housing of growing rabbits is associated with welfare concerns. An alternative system that has already been introduced involves pens with non-wire floors. An important aspect of group pens, for which the best solution has not yet been clearly demonstrated, is the choice of floor material. We investigated effects of two ground floor types—slatted plastic floor versus concrete floor with straw litter—on health-related parameters and weight of rabbits reared in large group pens on a commercial rabbit farm, i.e., with preventive coccidiostatic, and if required, additional therapeutic medical treatment. Pens were identical in dimensions, equipment (including platforms), and initial group size (60 animals/pen). Four pens were studied per ground floor type in three consecutive rearing periods, i.e., 12 pens/ground floor type. A higher percentage of rabbits per pen had clean fur if reared on straw. No significant differences were found in parasitic burden, mortality, pathological alterations, or causes of loss. Thus, often-expressed concerns that parasitic load and mortality would be higher in groups kept on straw were not confirmed when rabbits were housed under otherwise equal conditions. Average slaughter weight was higher in rabbits reared on a slatted plastic floor, confirming previous findings of a negative impact of straw litter on weight gain.

**Abstract:**

Cage housing of growing rabbits is associated with welfare concerns. An alternative system that has already been introduced involves pens with non-wire floors. An important aspect of group pens, for which the best solution has not yet been clearly demonstrated, is the choice of floor material. The study investigated effects of two ground floor types—slatted plastic floor versus concrete floor with straw litter—on health-related parameters and weight of rabbits reared in large group pens on a commercial rabbit farm, i.e., with preventive coccidiostatic, and if required, additional therapeutic medical treatment. Pens were identical in dimensions, equipment (including platforms), and initial group size (60 animals/pen). Four pens were studied per ground floor type in three consecutive rearing periods (in total, 12 pens per floor type). A higher percentage of rabbits per pen had clean fur if reared on straw (*p* < 0.05). No significant differences were found in the load of coccidial oocysts in collective faecal samples, mortality, pathological alterations, or causes of loss (*p* > 0.05). Thus, often-expressed concerns that parasitic load and mortality would be higher in groups kept on straw were not confirmed when rabbits were housed under otherwise equal conditions. Average slaughter weight was higher in rabbits reared on a slatted plastic floor (*p* < 0.05), confirming previous findings of a negative impact of straw litter on weight gain.

## 1. Introduction

To date, most rabbits reared for meat production in Europe are still housed in pairs or small groups of four to six animals in cages on wire net floors [1,2]. However, cage housing can have serious negative effects on rabbit welfare, for instance in terms of impaired behaviour [3,4,5]. There is an increasing societal and consumer demand for welfare-friendly products and rearing techniques [2,6], on the one hand presenting possibilities for brand development with economic benefits, and on the other hand pressure for changes in animal welfare legislation. There have been trends in some European countries of banning cage housing on wire-net floors, or at least of adopting minimum legal welfare requirements [2] (e.g., Austria, Germany, the Netherlands). However, rabbits are “under-represented in welfare research” and there are still “knowledge gaps that make political decisions difficult” [7]. Alternative systems that have already been introduced include pens with non-wire floors. These could benefit rabbit welfare because they avoid the presumed physical discomfort of wire floors and allow for more natural locomotor behaviour. However, it was pointed out that there is still a lack of knowledge regarding the equipment used for pen housing [2]. One important issue is the type of floor to recommend—the floor is among the most important technical elements of a housing system, as animals spend most of their time in contact with it [8]. Pens can have non-perforated or perforated floors (e.g., plastic slats or mesh). The latter help to separate animals from manure. On non-perforated floors, litter (e.g., straw) can be provided and is sometimes legally required. Views about straw-littered floors are controversial. It is generally assumed that straw can be beneficial to farm animal welfare, for example improving physical comfort when used as bedding [9]. Straw can also stimulate and allow the performance of exploratory, chewing, and foraging behaviours. A higher risk of infections with certain pathogens, increased labour, incompatibility with manure drainage systems, and higher costs are listed among disadvantages in pig and cattle housing [9]. In spite of potential advantages of littered floors, a higher percentage of rabbits was counted on non-littered wire floors than on wire floors covered with straw litter in preference tests [10,11], but ambient temperature also has an impact on floor preferences [8,12].

Contact of rabbits with other rabbits and their manure is considered a risk factor for coccidiosis and other diseases [13]. Coccidiosis has serious negative effects on health, decreases performance, and can increase mortality considerably [14,15]. Intestinal coccidiosis also seems to favour colonisation by other pathogens, such as *Escherichia coli* [15]. Impaired health due to coccidiosis, as well as higher mortality, were reported for rabbits housed on straw ([16] (the first out of two experiments), [17]). In addition, there are findings of impaired growth performance in rabbits housed on straw compared with other systems, which were partly attributed to a reduced feed intake due to the consumption of bedding material and partly to coccidiosis [18]. Increased physical activity might also explain a reduced growth rate. For instance, higher percentages of locomotor and comfort behaviours were shown in rabbits housed in a pen with a straw-littered floor compared with a pen with wire-netting only [17]. As a result, straw litter is sometimes considered unsuitable and a risk to growth performance and animal welfare [2]. To our knowledge, only three studies [8,10,19] compared effects of perforated versus littered floor types on performance, activity, or health and mortality while controlling for other factors, such as group size and stocking density; other studies compared whole housing systems [16,17,20], and thus do not allow one to draw conclusions about the impact of floor type alone. Morisse et al. [10] investigated time budgets, comparing groups housed in pens with wire-net floors with groups housed in pens partly littered with straw, while controlling for other factors. They reported more time allocated to activities such as feeding and social behaviours in the all wire-net pens but no differences in other activities, such as comfort, exploratory, and locomotor behaviours. Overall activity was not reported.

For three experiments, including groups housed on straw litter, either the occurrence of coccidiosis according to necropsy or oocystal counts and mortality were reported with regard to floor type ([10,16] (first and second experiment)); two of them did not show a higher parasitic load or mortality when housing rabbits on straw litter compared with wire systems with [16] (second experiment) and without prophylactic medication [10]. Gerencsér et al. [8] found no significant difference in mortality in rabbits from an experimental farm reared in groups of 14 animals in identical pens on either wire mesh, plastic mesh, or wire-mesh floor with straw litter with prophylactic medication, although they reported quite large numerical differences in mortality rates between floor types, with the highest mortality on straw litter. To our knowledge, this is the only published study comparing the effects of a perforated plastic floor and straw litter on the mortality of growing rabbits without varying other factors, such as group size, stocking density, or enclosure dimensions. Yet, only three pens per floor type were monitored, and especially with regard to health and infection pressure, pen effects can be large because of contagion of pen mates. Also, the study did not investigate larger groups, although they are found in commercial farms. Increasing group size could increase infection pressure and mortality, and this effect is feared, especially with straw litter [17,21]. While mortality and coccidiosis have received some attention, to our knowledge detailed causes of loss in growing rabbits according to necropsy have not been compared between different ground floor types so far.

Although concrete flooring with straw litter is a common floor type, the previous controlled studies have investigated wire-mesh flooring littered with straw [8,10,19]. Thus, the aim of our study was to test effects of ground floor type (slatted plastic versus straw-littered concrete floor) on health-related parameters, live weight before slaughter, and activity of rabbits reared in pens in large groups under conditions found on commercial rabbit farms. The conditions were designed according to the minimum legal requirements for the housing of growing rabbits in Austria, and thus differed from previous studies in that the stocking density was lower and platforms were provided in all pens. In order to isolate the effects of the type of ground floor, the pens were located in the same environment, managed alike (including medical treatments), and were identical in dimensions, equipment (including number and type of platforms), and initial numbers of animals, i.e., stocking density.

## 2. Animals, Materials and Methods 

The study was approved by the Ethics Committee of the University of Veterinary Medicine, Vienna, in accordance with guidelines for Good Scientific Practice and with national legislation.

### 2.1. Animals and Housing

The experiment was carried out on a commercial Austrian rabbit farm (in Lower Austria, near the city of Amstetten) that kept breeding does and growing rabbits. The region is dominated by a warm temperate climate. The annual mean temperature is 8.6 °C and the mean annual precipitation amount is 843 mm/year. The growing rabbits involved in the study were hybrid animals born on the farm and offspring of breeding rabbits bought from the supplier Bauer, Heilbronn, Germany. The supplier’s animals were originally based on ZIKA animals. Clinically healthy, weaned rabbits aged 38 days were randomly assigned by the experimenters to single-sex pens with 60 animals per pen. Stocking density was 9.60 animals/m^2^ of ground floor or 6.57 animals/m^2^ including the elevated platforms in the pens. After a fattening period of 45 days (at 83 days of age) half of the animals per pen were slaughtered according to the farm’s usual practice in response to retailer demands. The remaining animals were slaughtered one week later. Animals were observed until the day before first slaughter (at 82 days of age). The procedure was the same in the three successive trials.

**Figure 1:**
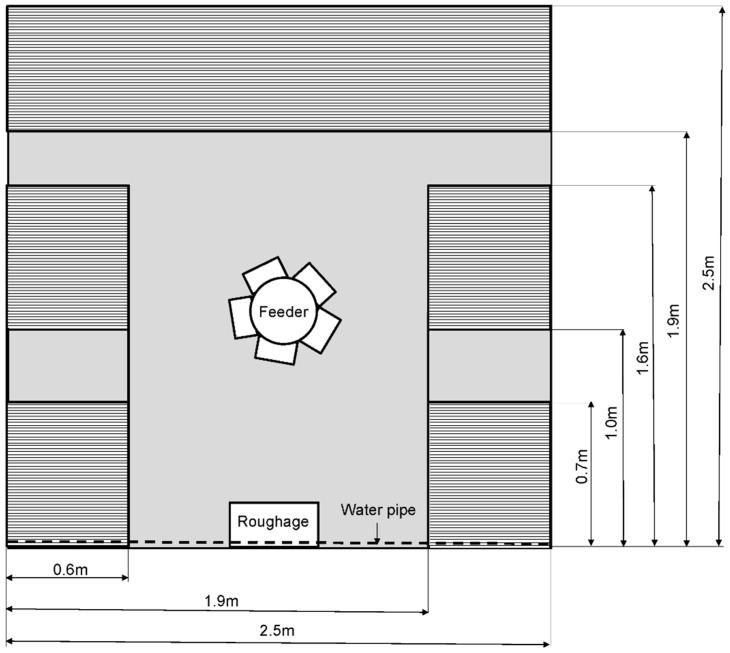
Schematic drawing of a pen seen from above. Ground floor depicted in grey, with elevated platforms depicted as striped. The ground floor consisted either of concrete littered with straw (Straw pens) or of slatted plastic panels (Slat pens).

All trials took place in the first out of three compartments of a growing rabbit barn. The rabbits were kept in open-top pens, with the ground floor consisting either of concrete littered with straw (Straw pens: four pens/trial) or of slatted plastic panels (Slat pens: four pens/trial). All eight pens were located in the same compartment; the Straw and Slat pens were only divided by a working corridor. In Straw pens, litter was not removed or exchanged during the fattening period but additional straw was added daily to prevent the development of damp areas. Slat pens contained panels designed for farrowing piglets with a distance of 10 mm between slats and were raised to provide space for the manure under the floor. Manure accumulating under these pens was removed twice per week with a scraper. Pen walls (105 cm high) consisted of a lower section of 60 cm of plastic panels topped by an upper section of 45 cm of wire netting. Each pen measured 6.25 m² (2.5 × 2.5 m) and contained five raised platforms attached to the walls on three sides of the pen, at a height of 30 cm from the floor of the pen, made from slatted plastic panels, such as ground floor panels in Slat pens. On both the left and right side of the pen there were two platforms, each measuring 0.57 × 0.65 m, connected by a metal rail. The fifth platform along the rear side of the pen measured 2.5 × 0.57 m. Each pen had 2.9 m^2^ of raised platforms in total. The roughage rack inside the pen was attached to the front door, which was made of wire netting. Each pen had a manually filled feeder for the pelleted diet in its centre, five nipple drinkers, and two wooden gnawing bars attached to the side walls. For a schematic drawing of a pen seen from above, see Figure 1. Straw, a standard pelleted fattening diet, and fresh water were offered ad libitum. Animals were fed a diet for weaners until the age of eight weeks, followed by a diet for fattening rabbits until slaughter. Neither the Straw nor the Slat pens were cleaned during a trial. Before each trial, the pens were prepared by removing litter and gnawing materials of previous rabbit groups, thorough cleaning, and disinfection. There was an all-in, all-out management approach within the compartment. However, to enter the second and third compartments, caretakers had to pass the first compartment. All animals received a coccidiostat (robenidine or salinomycin, implemented in a rotation programme by the farm) prophylactically in the pelleted diet until the withdrawal period (5 days before slaughter), in line with standard practice on the farm. During the withdrawal period before slaughter, a diet without coccidiostat was given. The health status of the animals was monitored daily by the caretakers. In the case of increasing incidences of disease or mortality, the rabbits were examined by veterinarians and all animals were given antiparasitic and/or antibiotic treatment via the water supply. Such therapeutic treatment was necessary four times during each of the three trials. Antiparasitic treatment was administered once per trial. In trial 1 and trial 3, it was administered simultaneously with an antibiotic treatment, while in trial 2, without simultaneous antibiotic treatment. Within a trial, the medication was administered equally to rabbits in both pen types to ensure the comparability under the same conditions and to avoid further spread of infection. No conclusions about potential differences with and without medication can be drawn. However, we were interested in potential ground floor effects under commercial conditions, where prophylactic and therapeutic treatment is often used. Besides, it would have violated legal and ethical guidelines not to treat the animals.

The rabbits had a natural photoperiod with daylight entering through windows on both sides of the barn. The barn compartment used for the study had five windows of 80 cm × 80 cm. Additionally, artificial light was provided via 40-watt lamps. The daily lightening period was 14 h (controlled automatically). The trials were carried out between July and December. Each barn compartment, including the compartment where the three trials took place, had a separate forced ventilation system (negative pressure ventilation) and a central heating system. Ammonia content in the air, measured by a gas detector pump at the head level of the animals, was 12.7 ppm ± 10.6 ppm across the three trials. Average temperature across all trials was 18.9 °C ± 4.6 °C.

### 2.2. Allocation of Animals to Treatments

In order to conduct an experiment with 12 rabbit groups for each ground floor type, three successive trials in three consecutive rearing periods were necessary due to space limitations. For the sake of simplicity, we will discuss 12 pens per condition of ground floor type for the remainder of the article. During each of the three successive trials, eight rabbit groups were monitored (four on straw litter, four on slatted plastic floor, two floor types per sex). A total of 1440 rabbits in 24 groups were included in the three trials; 360 animals of each sex were studied in six pens on each of the two floor types.

### 2.3. Recorded Parameters

#### 2.3.1. Soiling of Pens and Animals and Hind Leg Condition

At the end of each trial, when rabbits were aged 82 days, the pen floor surface (ground floor and platform surface) was evaluated by a trained observer standing in front of each pen. The observer during the first trial differed from the observer during the second and third trials. The surface was categorised as (1) dry or wet (obviously moist), and (2) as either slightly (<30%), moderately (30–50%), or heavily (>50%) soiled, i.e., covered with smears of droppings or a thin film of faecal material in case of the Slat pens, or covered with droppings in case of the Straw pens. The degree of soiling of individual animals was evaluated by examining their hind legs and perirectal area during final weighing, using a 4-point score used by Tillmann et al. [22] (Table 1). Soiling of the perirectal area was included in the score, but signs of diarrhoea were also noted separately on the scoring sheet. During the first trial two trained veterinarians scored the animals, balanced for ground floor type. During the second and third trial only one of them scored all the animals. On average 30 ± 1.6 randomly selected rabbits per pen (in total 723 animals) were examined. Based on the examined animals, the percentage of clean, slightly, moderately, or heavily soiled rabbits per pen was calculated. During the examination, the animals were also examined for signs of pododermatitis.

#### 2.3.2. Parasitic Load

Over the course of each fattening period, a total of four samples of faeces were collected per pen and examined for coccidial oocysts (in the first, second, and fourth week of fattening, as well as on the day before first slaughter, i.e., at the beginning of the seventh week when the rabbits were aged 82 days). At each sampling time, at least 30 g of manure was collected from each pen. Faeces were collected by placing three plastic shoe trays in each pen overnight and then picking faeces randomly from different areas of the trays in the morning. The trays had aluminium frames to prevent the rabbits gnawing at the plastic and drain holes to allow urine to drain off. 

First, all samples were analysed for parasitic objects by means of a combined sedimentation-flotation technique with concentrated sugar solution (specific gravity 1.28). If the results of this test were positive, the amount of oocysts/g faeces (OPG) was additionally counted using a modified McMaster technique with a minimum detection level of 50 OPG. In instances where oocysts were detected by the sedimentation-flotation procedure but McMaster counting results were negative, a number of 10 oocysts/g faeces was adopted for data analysis. The different *Eimeria* spp. were not specified, since all rabbits had the same origin and were housed in the same environment. 

#### 2.3.3. Mortality and Post-Mortem Examinations

Mortality rates were calculated by relating the number of losses per pen to the initial group size. Over the course of the three trials, 131 rabbits of the 720 rabbits from straw-littered pens and 103 of the 720 rabbits from slatted plastic floor pens were found dead or were moribund and had to be culled. To assess the causes of loss, 64 rabbits (30 from slatted plastic floor pens and 34 from straw pens, corresponding to 29.1% and 26.0% of lost animals, respectively) were subjected to necropsy and histological examinations, performed by one blinded veterinarian pathologist. The animals were selected to match the time of death as closely as possible but randomly distributed over pens within the same system. Intestinal samples of all 64 necropsied rabbits were analysed for coccidial oocysts using a combined sedimentation-flotation technique. Bacteriological and mycological examinations (in total 36) were only requested in cases of suspected infections of the respiratory or digestive tract. Whenever bacteriological examinations were requested by the pathologist, antibiograms were also ordered. The results were always forwarded to the farm owner and to the veterinarian who advised the farmer. The main pathological findings were considered the cause of loss (either of death or in case of moribund animals the cause for culling). They were classified according to the body systems affected, i.e., respiratory system, digestive tract, or both in case of coincidence of digestive and respiratory tract diseases that could have both caused the loss. Respiratory affections included primarily bacterial infections, for example caused by *Pasteurella multocida* or *Bordetella bronchiseptica*. Diseases of the digestive system often consisted of bacterial infections (e.g., *Clostridium* spp., *Escherichia coli*) or intestinal coccidiosis. In one case it was not possible to assign the main finding to the respiratory or digestive tract (i.e., interstitial nephritis), thus it was categorised as “miscellaneous”. 

To shed light on general health problems, regardless of lethality, overall pathological findings, i.e., both main and secondary pathological findings, were categorised according to the altered organ or organ system, for instance diseases of the lower respiratory tract (tracheitis, bronchitis, pleuritis, pneumonia). Assignment to more than one category was possible to describe animals with two or more pathological alterations.

#### 2.3.4. Weight Data

All rabbits were weighed individually at the beginning of the trials when placed in pens to calculate the initial average body weight per pen. At the end of each trial, when aged 82 days, the rabbits randomly selected and examined for soiling were weighed to calculate the average live weight per pen shortly before slaughter.

#### 2.3.5. Activity Analysis

In order to examine causes of a potential ground floor effect on weight, the animals’ activity was recorded via a video surveillance system (Multieye GreenWatch NVR recorder, Artec technologies AG, Diepholz, Germany). An infra-red light-sensitive video camera (Acti^®^, type ACM 1431P) and infra-red lights (from VideoSecur^®^, type IR-LED294S-90 LED) were installed above every pen before animals were placed into pens. For every rabbit group, 24 h of video material was collected on day 81 (the penultimate day before slaughter). Video data over the whole 24 h were analysed using scan sampling by a trained observer. Due to technical problems, it was not possible to analyse the videos of four groups over the whole duration of 24 h (one male group per floor type and two female groups on slatted plastic floor), resulting in a sample size of 20 groups. In intervals of 30 min, the observer paused the video stream and counted the number of active animals visible on all platforms and the floor, resulting in 48 scans per pen. Animals were rated as “active” when sitting vigilantly, in an upright position with straightened front legs, or when engaging in behaviours such as feeding or foraging, locomotor, comfort, exploratory, or social behaviours. Since other animals could theoretically block the view (e.g., when at the time of the scan one animal would pass in front of another), per definition at least 40% of a rabbit’s body had to be visible in order to count it. In cases of doubt, the seconds before or after the sample point were analysed. Based on these scans, the mean percentage of active rabbits per pen was calculated in MS Excel (Microsoft Office 2010). 

### 2.4. Data Analysis

Data were analysed and graphically presented using the statistical software package IBM SPSS Statistics for Windows, Version 25.0 (IBM Corp., Armonk, N.Y., USA). Data are provided as Supplementary Materials (Data S1: data_windschnurer.et.al.xlsx). Except for pathological alterations and causes of loss, all parameters were analysed at pen level, i.e., the group was considered the statistical unit. Soiling or wetness of pens, pathological alterations, as well as causes of loss (nominal and ordinal data) and their possible dependence on ground floor type or trial were investigated using crosstabs, Chi-square, and Fisher’s exact test. Standardised residuals were calculated to help identify cells with meaningful differences (standardised residuals |>1|). Analyses of the parasitic burden were based on ratio calculations of McMaster results. The first sampling, taken in the first week of fattening, was used as baseline in order to take the initial parasitic burden into account by dividing the number of oocysts in the following three samplings by the respective number of oocysts of the first sampling, resulting in ratios of 2:1, 3:1, and 4:1. In one baseline sample (from a pen with straw litter), no coccidia were found. For this sample, a value of 0.1 oocysts/g faeces (OPG) was adopted for the ratio calculations because a division by zero is not possible. Non-parametric Mann-Whitney U tests were run to test for differences in the ratios between floor types, since the data did not fit a normal distribution (according to Shapiro-Wilks tests). Kruskal-Wallis tests were used to test for differences in the ratios between trials, with Mann-Whitney U tests as post hoc tests in cases of a significant difference. Initial average body weights, and percentages of clean, moderately, and heavily soiled rabbits/pen were compared between ground floor types using Mann-Whitney U tests, and between trials using Kruskal-Wallis tests, with Mann-Whitney U tests as post hoc tests. For mortality, average slaughter weight/pen, and the percentage of slightly soiled rabbits/pen general linear models with floor type, trial, and their interaction as fixed effects were calculated. Equality of variances was given in all instances according to Levene’s test. For the percentage of active animals/pen, a general linear model with floor type and sex as fixed effects was calculated after a sinus transformation of the activity data due to a violation of homoscedasticity (Levene’s test). It was controlled for sex because activity could not be analysed for the same number of female groups per floor type. Due to violation of homoscedasticity (Levene’s test), trial number could not be included in the model. Since activity data were not available for the same number of female and male groups per trial, two univariate analyses of variance were performed, once for male and once for female groups, with trial number as the fixed effect. Untransformed data are presented in the results section; due to very large outliers, ratio values of parasitic burden are depicted on a logarithmic scale. Differences with *p* ≤ 0.05 are referred to as significant, with *p* ≤ 0.1 as a trend.

## 3. Results

### 3.1. Health-Related Parameters

#### 3.1.1. Soiling and Wetness of Pens

All 12 Straw pens were moderately soiled. Nine Slat pens were assessed as moderately and three as heavily soiled. However, no overall effect of ground floor type on soiling was found (Fisher’s exact test: *p* = 0.22, n = 24). One pen was assessed as heavily soiled during trial 1, and two pens were assessed as heavily soiled during trial 3. There was no association between the degree of soiling of pens and trials (χ^2^ = 2.29; df = 2; *p =* 0.32, n = 24). During the first trial two Slat pens and one Straw pen were rated as wet. Floor surface wetness was found not to depend on ground floor type (Fisher’s exact test: *p* > 0.99, n = 24) but on trial number (χ^2^ = 6.86; df = 2; *p =* 0.03, n = 24).

#### 3.1.2. Soiling of Animals and Hind Leg Condition

**Figure 2:**
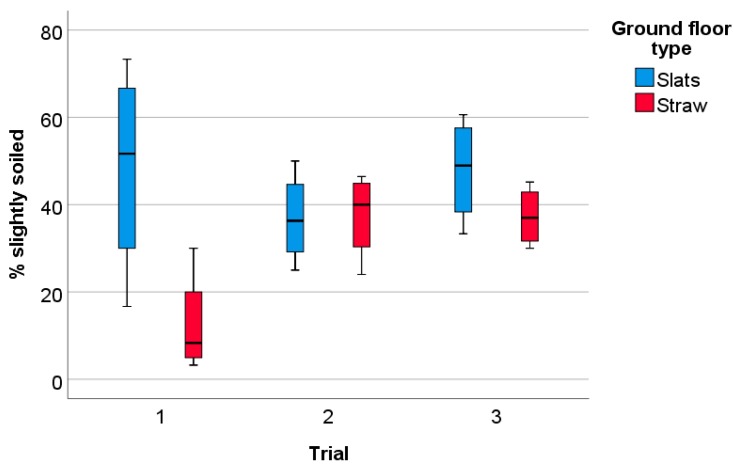
Percentages of slightly soiled animals per pen in Slat pens (ground floor consisting of slatted plastic panels, n = 4 per trial) and Straw pens (pens with the ground floor consisting of concrete littered with straw, n = 4 per trial).

Overall, there was a large variation in the degree of soiling between pens within both ground floor types (Table 2). At the end of the fattening period, a higher percentage of clean animals was found in Straw pens (Table 2; U = −2.17, *p* = 0.03). There was no significant difference between Slat and Straw pens in percentages of moderately or heavily soiled animals (Table 2; U = −1.43/−1.14, *p* = 0.15/0.25). However, there was a significant effect of ground floor type (F = 7.35, *p* = 0.01) on the percentage of slightly soiled rabbits and an interaction of ground floor type with trial number (Figure 2; F = 3.67, *p* < 0.05). 

**Figure 3:**
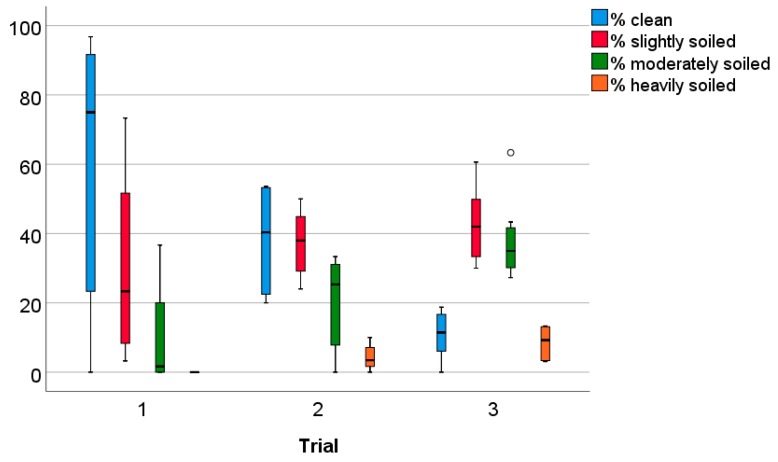
Soiling of animals (percentages of clean, slightly, moderately, or heavily soiled animals per pen) across the three trials, independently from ground floor type; n = 8 pens per trial.

Additionally, significant differences in the percentages of clean, moderately, and heavily soiled animals were found across the three trials (Figure 3; Kruskal-Wallis tests: % clean: H = 9.71, df = 2, *p* = 0.01; % moderately soiled: H = 10.84, df = 2, *p* < 0.01; % heavily soiled: H = 14.49, df = 2, *p* < 0.01). A lower percentage was heavily soiled in trial 1 compared with trial 2 (Mann-Whitney U test: Z = −2.90, *p* < 0.01). A trend for a higher percentage of clean animals and significantly lower percentages of moderately and heavily soiled animals in trial 1 compared with trial 3 were found (% clean trial 1 vs. trial 3: Z = −1.95, *p* = 0.05; % moderately soiled: Z = −2.91, P < 0.01; % heavily soiled: Z = −3.60, *p* < 0.01). Comparing trial 2 and trial 3, higher percentages of clean animals and lower percentages of moderately soiled animals were found in trial 2 (% clean: Z = −3.37, *p* < 0.01; % moderately soiled: Z = −2.47, *p* = 0.01). Signs of diarrhoea were only observed in one animal that had been reared in a Straw pen in trial 3. Pododermatitis was never observed.

#### 3.1.3. Parasitic Load

**Figure 4:**
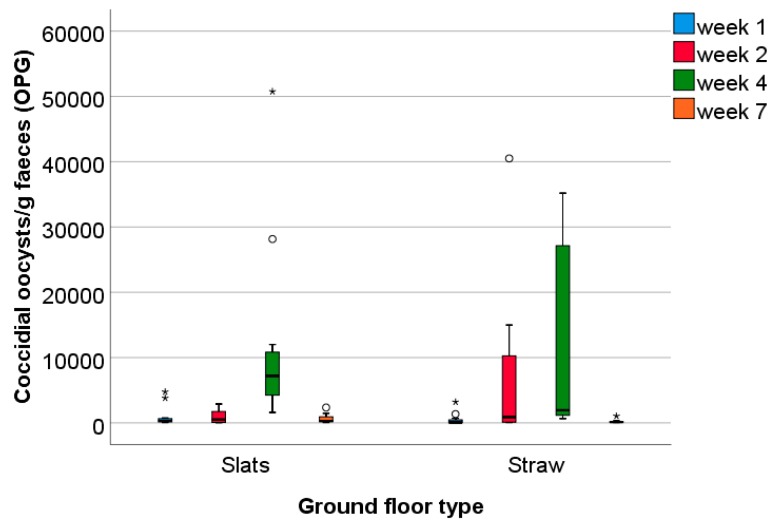
Number of coccidial oocysts/g faeces (OPG) in pens with the ground floor consisting of slatted plastic panels (n = 12) and in pens with the ground floor consisting of concrete littered with straw (n = 12); sampling times: first, second, and fourth week of fattening, as well as the day before first slaughter of the growing rabbits at the beginning of week 7 when aged 82 days.

**Figure 5:**
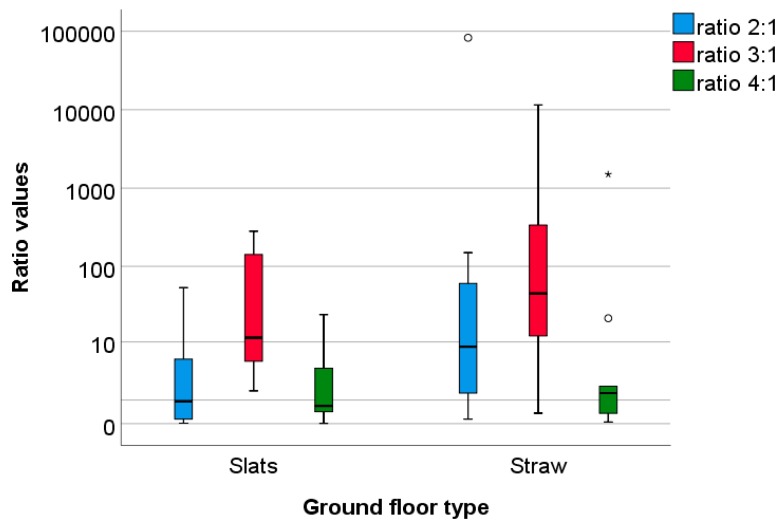
Ratio values calculated for monitoring the parasitic burden. Due to very large outliers, values are depicted on the logarithmic scale. Faecal samples from pens with the ground floor consisting of slatted plastic panels (n = 12) or pens with the ground floor consisting of concrete littered with straw (n = 12) were analysed for numbers of oocysts/g faeces at four sampling times (1–4), corresponding to the first, second, and fourth week of fattening, as well as the day before first slaughter of the growing rabbits at the beginning of week 7. The number of oocysts/g faeces in the first sampling (1) was taken as the baseline in order to take the initial parasitic burden into account. The number of oocysts at the following samplings (2, 3, and 4, in the second, fourth, and seventh week of fattening) was divided by the number of oocysts/g faeces in the first sampling (1), resulting in ratios of 2:1, 3:1, and 4:1.

**Figure 6:**
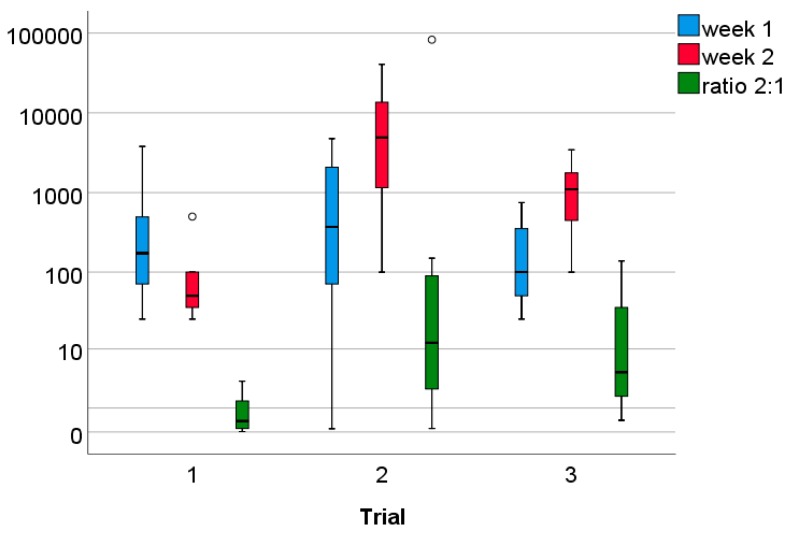
Parasitic burden in the three trials in week 1 and week 2 (coccidial oocysts/g faeces), as well as ratio 2:1 (number of oocysts at the sampling in the second week of fattening, divided by the number of oocysts/g faeces at the sampling in the first week), regardless of ground floor type. Due to very large outliers, values are depicted on the logarithmic scale; n = 8 pens per trial.

There was a large variation between pens in the number of coccidial oocysts/g faeces at the four sampling times (Figure 4). At the first sampling, the number of coccidial oocysts/g faeces tended to be higher in Slats pens (Slat pens: mean ± SD: 963 ± 1575, median: 300 oocysts/g; Straw pens: 496 ± 939, median: 75 oocysts/g; U = −1.68, *p* = 0.09). The following analyses were based on ratio calculations to take initial parasitic burdens into account. The numbers of oocysts at the three later samplings were divided by the number of oocysts in the first sampling. Ratio values did not differ significantly between Slat and Straw pens for any sampling point (ratio 2:1, U = −1.85, *p* = 0.07; ratio 3:1, U = − 0.98, *p* = 0.33; ratio 4:1, U = −0.06, *p* = 0.95, n = 24, see also Figure 5) but the ratio 2:1 values of Slat pens tended to be smaller due to a smaller increase in coccidial oocysts from the first to the second sampling compared to Straw pens (see Figure 4). Regarding potential differences between trials, there were significant differences only for ratio 2:1 values (H = −7.70, df = 2, *p* = 0.02). Post hoc testing revealed that ratio 2:1 values were smaller during trial 1 than during trial 2 and trial 3 (Figure 6; trial 1 vs. 2: Z = −2.21, *p* = 0.03, trial 1 vs. trial 3: Z = −2.52, *p* = 0.01, trial 2 vs. trial 3: Z = −0.63, *p* = 0.53). This is due to an increase in coccidial oocysts from the first to the second sampling in trial 2 and trial 3, which did not occur in trial 1.

#### 3.1.4. Mortality, Causes of Loss, and Overall Pathological Findings

**Figure 7:**
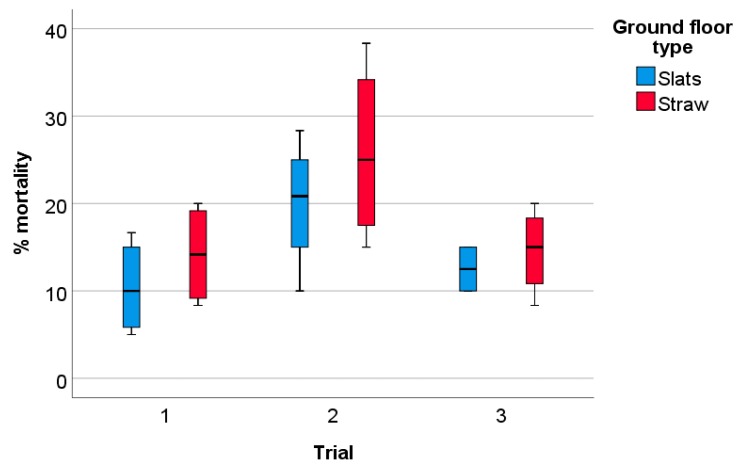
Mortality at pen level according to ground floor type and trial. Slats: pens with ground floor consisting of slatted plastic panels, n = 4 per trial; Straw: pens with the ground floor consisting of concrete littered, n = 4 per trial.

There was no significant effect of ground floor type (F = 2.07, *p* = 0.17) and no interaction between ground floor type and trial (F = 0.16, *p* = 0.85), but a significant effect of trial (F = 6.15, *p* = 0.01) on the number of losses per pen (dead animals or moribund animals that had to be culled) before slaughter, at 82 days of age (Figure 7). Across the three trials, mortality was 14.3% ± 6.7% (mean ± SD) in Slat pens and 18.2% ± 8.8% in Straw pens. According to post hoc testing, the mortality was significantly higher in trial 2 compared with trial 1 and trial 3 (trial 1 vs. trial 3: *p* > 0.05, trial 2 vs. trial 1 and trial 3: *p* < 0.05). Across ground floor types, mortality was 12.3 % ± 5.6% in trial 1, 22.9% ± 9.0% in trial 2, and 13.5% ± 3.9% in trial 3.

Digestive disorders were the main cause of loss. Respiratory problems were never solely the main cause of loss but were always found in combination with digestive disorders. There was no relationship between ground floor type and diagnosed causes of loss attributed either to the digestive, both respiratory and digestive tract, or others (χ^2^ = 1.20; df = 2; *p* = 0.55; Table 3). No relationship was detected between ground floor type and overall pathological findings, i.e., both main and secondary pathological findings (*p* > 0.05, Table 4).

Causes of loss and overall pathological findings differed partly across the three trials (Table 5). Health issues related to the respiratory tract were more common during the first trial. 

### 3.2. Weight and Activity

**Figure 8:**
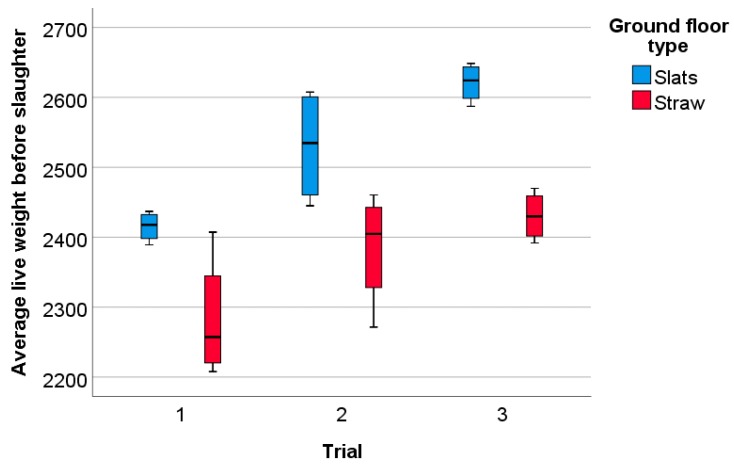
Average live weight (g) shortly before slaughter in g at 82 days of age, according to ground floor type and trial. Slats: pens with ground floor consisting of slatted plastic panels, n = 4 per trial; Straw: pens with the ground floor consisting of concrete littered with straw, n = 4 per trial.

Weaned rabbits placed in Slat pens (n = 12) or Straw pens (n = 12) at 38 days of age did not differ in initial average body weight (1018 ± 79.7 g (Slat pens) vs. 1011 ± 74.8 g (Straw pens); U = −0.29, *p* = 0.77). However, initial average body weight differed across the trials (trial 1: 944 ± 11.2 g, trial 2: 988 ± 28.8 g, trial 3: 1112 ± 23.7 g, H = 19.28, df = 2, *p* < 0.01). Average initial weight was lowest in trial 1 and highest in trial 3 (trial 1 vs. trial2: Z = −2.94, *p* < 0.01; trial 3 vs. both trial 1 vs. and trial 2: Z = −3.36, *p* < 0.01). Rearing rabbits on a slatted plastic rather than a straw-littered ground floor resulted in a significantly higher average live weight shortly before slaughter at 82 days of age (2522 ± 99.5 g vs. 2366 ± 92.2 g; F = 36.91, *p* < 0.01). There was also a significant effect of trial on average live weight before slaughter (F = 16.04, *p* < 0.01), but no interaction between ground floor and trial (F = 0.47, *p* = 0.63). According to post hoc testing, average final weights were lower in trial 1 compared with the other two trials (Figure 8; trial 1 vs. trial 1 and trial 3: *p* < 0.05, trial 2 vs. trial 3: *p* > 0.05).

There was no significant effect of ground floor type (F = 1.00, *p* = 0.33) or sex (F = 0.56, *p* = 0.46) on the percentage of active rabbits per pen, nor an interaction of ground floor type with sex (F = 0.01, *p* = 0.92) (Slat pens, male: mean ± SD: 26.03% ± 5.43%, n = 5, female: 29.09% ± 2.48%, n = 4; Straw pens, male: 30.07% ± 4.52%, n = 5, female: 28.95% ± 1.67%, n = 6). Trial number did not have any effect on activity, neither within male nor within female pens (male: F = 0.72, *p* = 0.52, trial 1: 20.88% ± 1.36%, n = 3, trial 2: 31.05% ± 1.68%, n = 3, trial 3: 31.18% ± 1.91%, n = 4; female: F = 2.50, *p* = 0.15, trial 1: 27.53% ± 0.19%, n = 2, trial 2: 28.46% ± 2.24%, n = 4, trial 3: 30.29% ±1.32%, n = 4).

## 4. Discussion

### 4.1. Effect of Ground Floor Type on Health-Related Parameters

#### 4.1.1. Degree of Soiling

Soiling of animals and pens due to contact with manure is presumably linked with a higher probability of contagion with certain pathogens. Regarding the soiling score for individual animals, the idea was that the ground floor type might not only affect the degree of soiling of animals via direct contact with the floor, but also indirectly via an impact on animal health. For instance, increased parasitic burden might lead to diarrhoea, and ill animals might show less grooming behaviour. 

Higher percentages of clean animals were found in Straw pens, while higher percentages of slightly dirty animals were found in Slat pens. This agrees with the rating of some Slat pens as being heavily soiled, which was not observed in any Straw pens, although no significant difference in the average degree of soiling or wetness of the pens was found. Pen soiling was assessed on a three-point scale, while individual animals were assessed on a four-point scale, which may show higher sensitivity. This may be a reason why the percentage of clean animals in Straw pens varied widely, although all these pens were assessed as moderately soiled. Thus, more accurate information on soiling and its effects on the animals may be gained by looking at individual animals.

To our knowledge, soiling of growing rabbits kept on litter or their pen surfaces has never been assessed systematically, although it has been suggested that straw litter becomes soiled quickly [17]. Morisse et al. [10] speculated that rabbits preferred the cleanliness and dryness of the wire floor, since rabbits showed a preference for lying in the wire area of pens with both wire and straw litter, but no soiling of either pens or animals was recorded. 

The effects of the method of straw provision (in a rack versus loose) and types of elevated floors (slatted versus solid) on soiling were compared in a breeding doe system [13,23]. Parts of floor with straw litter and solid elevated platforms were described as rather dirty (on average 50% covered with droppings or smears, corresponding to our “heavily soiled” score). However, in the present study, Straw pens were never soiled more than moderately (30–50%), while only a few Slat pens were more heavily soiled (more than 50% of the inspected surface) than all other pens. Both ground floor types seemed to allow good drainage or absorption of urine, since only two Slat pens and one Straw pen were considered wet. One may argue that straw was also offered in Slat pens, so that it may have accumulated on the ground floor. However, there was rarely straw on the floor. The contrasting findings between the study of the breeding doe system and our study might be partly explained by different enclosure designs. The restricted littered area in the doe system might have induced animals to select it as a place for toileting. In the wild, rabbit groups tend to use latrine sites for elimination [24]. Besides, results between does and growing rabbits are difficult to compare, since droppings of does are larger, which could result in different soiling scores on the same type of slatted floor. Also, the distance between slats, or in cases of circular holes, their diameter and the overall percentage of perforation, must be considered in terms of soiling [22,25]. In our study, a distance of 10 mm between slats was used in accordance with Austrian legislation, while in Germany up to 11 mm are allowed for fattening rabbits. Larger distances might lead to less soiling. In addition, the stocking density, which was reduced compared to previous studies due to the presence of the additional platforms, can affect the degree of soiling. Other aspects that merit closer investigation are the type and quality of litter (e.g., straw length), the flooring beneath (e.g., wire net or concrete), and the management (intervals of adding or replacing straw and the amount of straw used). Regarding the management, neither the Straw nor the Slat pens were cleaned during a trial. The objective was to compare two ground floor types that are suitable for commercial conditions. When Slat pens were soiled in our experiment, there were not big accumulations of droppings that blocked the openings, but in fact a thin film or layer of faecal material. This material can only be removed by cleaning with high pressure and hot water, which is not possible while animals are in the system. In Straw pens, additional straw was added daily to prevent the development of damp areas, as was usual practice on this farm. This could explain why rabbits stayed cleaner in littered pens.

#### 4.1.2. Parasitism 

Parasitic load was monitored by faecal analysis. In addition, intestinal samples from all necropsies were examined for coccidial oocysts. Overall, parasitism was not significantly influenced by ground floor type. This agrees with the results of the second but not the first experiment published in the same article by Lambertini et al. [16], who compared cage housing in pairs on wire floors with group housing in straw-littered pens. In their second experiment, animals received a coccidiostat and additional prophylactic medication before the trial, while in the first animals had received a coccidiostat only. In the present experiment, the prophylactic and therapeutic treatment might have accounted for the lack of differences. However, Morisse et al. [10] also found no differences in oocysts/g faeces between all-wire pens and pens with part-wire and part straw-littered floors without any prophylactic or therapeutic medication. They reported a low mortality of 0.8%. In the present study, mortality was considerably higher, and thus the prophylactic and therapeutic treatment might not have been as effective as hoped. A more likely reason why coccidiosis was not affected by floor type was that the perforated floor did not result in a reduction of the parasitic burden by separating the rabbits sufficiently from manure. The fact that higher percentages of soiled rabbits were found in Slat pens supports this idea. 

Adult rabbits function as healthy carriers of coccidia [14] and the doe is the most common source of infection for young rabbits [26]. In the present study, the kits were most likely infected before weaning, and findings of oocysts in the first week of fattening support this assumption.

#### 4.1.3. Mortality, Causes of Loss, and Overall Pathological Findings

Mortality rates (animals found dead or moribund animals that had to be culled) were not related to ground floor type but were generally rather high, with averages of 14.3% (Slats pens) and 18.2% (Straw pens). High post-weaning mortality rates for growing rabbits were also found in other studies. In reviews, post-weaning mortalities between 2.4% and 43.1% [27], and 3.6% and 20.2% [5] are reported, indicating a large variation. For France, mortality rates around 8.5% are reported, but with frequent preventive anti-biotherapy. Still, mortality may frequently exceed 15% and reach up to 50% [28]. Regarding specific floor types, Princz et al. [21] found a post-weaning mortality of 11.4% in pens and cages (average data) and Gerencsér et al. [8] reported a rate of 6.7% for pens with a perforated plastic floor. For pens with straw litter, post-weaning mortality rates of 8.7%, 13.2%, 4.2–15.6%, and 35% were reported [8,16,17,20]. In addition to different severities of health problems and intensities of drug application, the studies included a variety of group sizes and stocking densities, which may have influenced infection pressure [17,29]. According to EFSA (European Food Safety Authority) [5], there has been no substantial decrease in mortality rates over the recent decades in spite of better hygiene standards, modernised rearing techniques, and improved environmental conditions. Also, the need for repeated medication for coccidiosis and other diseases is common in rabbit husbandry [5]. In the current study, antibiotics were only administered in terms of intervention but not routinely, although routine administration is a widespread practice, also noticed at the European Union level [30], supporting the need for refinement of systems.

The literature contains contradictory results on mortality in systems with straw litter compared with systems with perforated floors (wire mesh or plastic mesh or slats) [8,10,16,17]. One reason may be that some studies compared whole housing systems, so that besides floor type, other factors such as group size, stocking density, or enclosure dimensions varied simultaneously. To our knowledge, only two studies compared mortality on different floor types under otherwise equal conditions and did not find significant differences between wire-mesh pens littered with straw and wire-mesh only [8,10] or plastic-mesh pens [8]. Yet, Morisse et al. [10] had no treatment group on slatted plastic flooring. Gerencsér et al. [8] reported that the mortality rate in straw-littered pens (8.7%) was numerically almost twice as high as in wire-mesh pens (4.5%) and higher than in plastic-mesh pens (6.7%), but had a low sample size, with only three pens per treatment.

Contact with manure on solid floors and ingestion of litter contaminated with manure are considered risk factors for the transmission of pathogens and higher mortality, especially regarding enteric diseases, such as coccidiosis [5,17]. However, we found a numerically higher degree of soiling in Slat pens, as well as a significantly higher percentage of slightly soiled animals, which may have prevented a beneficial effect of perforated floors on microbial burden. This could explain why mortality did not differ between floor types. Another reason could have been that all matched groups received the same preventive and therapeutic medication. However, the other studies where only floor types varied also found no significant difference in mortality with and without medication, comparing pens with straw litter versus perforated floors [8,10].

Digestive disorders were the predominant cause of death or reason for culling in our study, followed by a combination of diseases of the digestive and respiratory tracts, comparable to previous studies [5,31]. Digestive disorders often have a multi-factorial aetiology. Nutritional imbalances, e.g., regarding the fiber content, increase the susceptibility of rabbits to health problems, especially digestive disorders [28]. In the present study, all rabbits were fed the same pelleted diet and were provided with roughage (straw) in racks. 

The main causes of loss did not differ between ground floor types; neither did general pathological findings (taking main as well as secondary findings into account). The most common pathological findings were emaciation, enteritis, and intestinal coccidiosis, followed by diseases of the lower respiratory tract. As for respiratory diseases, almost all rabbits carry *Pasteurella* spp. latently in their upper respiratory tract [5]. Environmental factors, such as dust, a detrimental microclimate, or harmful gases (e.g., ammonia), can play a vital role in the outbreak of rhinitis and other symptoms of pasteurellosis [5,32]. Since the pens were all located close together in the same barn and straw was offered as roughage in all pens, it can be assumed that the microclimate and dust concentration were similar in all pens. Microclimate and dust concentrations might differ in buildings with either pens littered with straw or pens with slatted ground floor. For instance, in buildings with slatted ground floors, dust load, and thus the incidence of respiratory diseases, might be lower. However, such a research question would require an epidemiological study. 

In the present study, ammonia content in the air sometimes reached high levels. A negative impact on the animals can, thus, not be excluded. The average was 12.7 ppm. In Austria, there are no legal regulations for ammonia levels for rabbits. In Germany, ammonia content in the air should not exceed 10 ppm and must not exceed 20 ppm in the long term.

Knowledge of causes of mortality is important from a health and welfare point of view and can support diagnosis and prevention of diseases, which can also have financial benefits [33]. Since our study was carried out in a commercial setting, the results have a high practical value for rabbit farmers. Health protocols have to be established, hygiene standards must be optimised, and more research into prevention is warranted.

### 4.2. Effect of Ground Floor Type on Weight and Activity

At the start of the trials, there was no difference in average initial body weight, but slaughter weight was lower in Straw pens. Our results agree with previous findings of lower weight gain in littered systems [18]. Lower weight gains were partly explained by increased roughage or straw consumption and resulting lower intake of concentrate [19,34]. Yet, also in pens littered with wood shavings—which rabbits do not ingest—the performance was lower than in cage systems but did not differ from straw-littered pens [16]. Thus, other factors must also be relevant. Since in the present study no difference was found in the numbers of coccidial oocysts—neither in collective faecal samples, nor in gut samples in the pathological examinations—parasitism cannot explain the lower live weight shortly before slaughter. 

Final body weight in rabbits reared on straw could be also negatively influenced by increased activity, such as locomotion. Dal Bosco et al. [17] observed more activity in a pen with straw litter compared with a pen with wire-netting, according to the data of 10 focus animals per pen. However, the study design did not allow exclusion of a group effect, as only two pens were compared. Investigating time budgets of groups housed in pens with wire-netting partly littered with straw and wire-netting only, Morisse et al. [10] found differences in time allocated to feeding and social behaviours but no differences in comfort, investigatory, or locomotor behaviours. In the present study, we found that overall activity levels on platforms and the ground floor did not differ significantly between groups housed in pens with a slatted plastic floor or concrete floor with straw litter, which did not differ in any other characteristics. No conclusions are possible with regard to animals under the platforms. They were likely often resting, i.e., not active, since there were no water or feeding resources and there was not ample space, e.g., for locomotor behaviours. Informal observations during our presence at the farm support this notion.

There are indications that activity decreases, whereas resting increases with age [35]. Since activity was only analysed at the age of 81 days, no information about earlier activity levels is available. It was not the aim of the present study to look at behavioural changes related to age but to compare potential effects of two different ground floors on activity levels close to slaughter, because they are more likely to affect body weight. 

In sum, lower consumption of concentrate, but neither a higher parasitic load nor a higher activity induced by the straw litter, seems to have caused lower body weight of animals kept in Straw pens. It would have been useful to also analyse the intake of concentrate, feed conversion, carcass weight, dressing percentage, and the weight of the full gastro-intestinal tract. However, this was outside the scope of the present study. From an economic viewpoint, it is important to address such aspects in future studies.

### 4.3. Trial Effects

Potential differences between the three trials were analysed, as considerable variation in health and mortality between batches of growing rabbits within farms has been reported [36,37]. Causes of mortality and overall pathological alterations did not differ between ground floor types but some of them differed between trials. One reason might be seasonal effects because the trials took place from July to December. In trial 2, mortality as well as the incidences of intestinal coccidiosis and of enteritis, as a trend, were higher. This might be due to different treatment schemes—in all trials, antiparasitic treatment was administered; however, in trial 2 no antibiotic treatment was administered simultaneously with the antiparasitic treatment, in contrast to trials 1 and 3. Intestinal coccidiosis seems to favour colonisation with pathogenic bacteria [15]. The percentage of clean animals was highest during trial 1. In trial 1, also fewer cases of intestinal coccidiosis were detected. This supports a potential link between soiling of animals and intestinal coccidiosis.

Our study was designed to test for effects of ground floor types, but we also tried to keep the conditions stable across trials. Nevertheless, two veterinarians scored the individual animals during trial 1, while only one of them scored all the animals during the following two trials. Since both had trained the scoring during pilot testing, it is unlikely that an observer effect caused the differences between trials. Additionally, for the scoring of the pens, an observer effect is unlikely due to prior training and a very simple score.

Trial effects on final weight shortly before slaughter may be explained by initial weight differences at weaning. This is in line with Lang [37], who found differences between trials in body weight at weaning and in final body weight, as well as a significant positive relationship between the two parameters. 

There is need for further research into influencing factors on health and mortality of growing rabbits, including potential seasonal effects. Epidemiological approaches could help to explain variability in mortality between and within farms by analysing potential influencing factors, such as housing, management, preventive and therapeutic treatment, as well as season. 

## 5. Conclusions

Our study is the first investigation of health problems in growing rabbits housed in large groups on ground floors of concrete with straw litter or of slatted plastic under the same housing conditions in a commercial setting with preventive and therapeutic treatment. Rabbits stayed cleaner in straw-littered pens. The degree of soiling was very likely influenced by the management, i.e., the daily addition of straw in pens with litter to prevent the development of damp areas. Parasitic load and mortality did not differ between pens with different ground floor types. Likewise, no differences were found regarding causes of loss and overall pathological alterations. Previous findings of the negative impact of straw litter on weight gain were confirmed but the effect was not caused by a higher incidence of coccidiosis or higher activity levels on straw. Our results, in line with those of other groups, also imply that further studies investigating pen systems are necessary to optimise conditions, for instance with regard to the high mortality rates often found in rabbit husbandry. It is vital to analyse potential influencing factors on growing rabbit health and mortality, including potential seasonal effects, using an epidemiological approach. This might further help to explain differences in mortality between and within farms.

## Figures and Tables

**Table 1:** Definitions of the soiling score for the examination of individual rabbits at the age of 82 days (see also Tillmann et al. [22]).

Score	Definition
Clean	Hind legs clean (dry, slight discoloration possible) and clean perirectal area
Light soiling	Hind legs slightly dirty, dry, without accumulations of faeces and/or straw sticking to them and clean perirectal area
Moderate soiling	Hind legs moderately dirty, dry, possibly slight accumulation of faeces and/or straw sticking to them that can be removed without loss of hair and clean perirectal area
Heavy soiling	(a) Hind legs extensively dirty (large areas are dirty due to accumulations of dried faeces and/or straw sticking to legs, not removable without loss of hair) (b) hind legs wet and dirty or (c) perirectal area soiled with soft faeces or diarrhoea

**Table 2:** Degree of soiling (measured in percentage of rabbits per pen being clean, slightly, moderately, or heavily soiled) in pens with the ground floor consisting of slatted plastic panels (Slats, n = 12) or pens with the ground floor consisting of concrete littered with straw (Straw, n = 12) at the age of 82 days.

Degree of Soiling (%)	Ground Floor Type								
	Slats				Straw				Sig ^1^
	Med ^2^	Min-Max ^3^	M ^4^	SD ^5^	Med	Min-Max	M	SD	
Clean	15.0	0–83.3	23.9	25.86	48.7	12.9–96.8	48.6	32.27	0.03
Slightly soiled	43.3	16.7–73.3	44.4	16.23	31.7	3.2–46.4	29.1	15.18	0.01
Moderately soiled	28.6	0–63.3	29.0	15.82	13.7	0–40.0	16.5	17.08	0.15
Heavily soiled	3.2	0–9.1	2.7	2.89	5.7	0–13.3	5.9	5.83	0.25

Note: ^1^ *p* values of Mann-Whitney U tests or main effect of ground floor in linear model; ^2–5^ Descriptive statistics: median, minimum-maximum, mean, standard deviation.

**Table 3:** Causes of loss, i.e., main pathological findings, of animals reared in pens with the ground floor consisting of slatted plastic panels (Slats, n = 30) or pens with the ground floor consisting of concrete littered with straw (Straw, n = 34) according to necropsy diagnoses—number and percentage of animals attributed to body systems. No relationship between ground floor type and causes of loss was found (χ^2^ = 1.20; df = 2; *p* = 0.55).

Causes of Loss Attributed to Body Systems	Ground Floor Type		Total
	Slats	Straw	
Digestive tract ^1^			
Number	17	19	36
% within system	56.7%	55.9%	56.3%
Digestive and respiratory tract			
Number	12	15	27
% within system	40.0%	44.1%	42.2%
Miscellaneous pathology ^2^			
Number	1	0	1
% within system	3.3%	0%	1.6%

Note: ^1^ Once in combination with absceding inflammation of a leg; ^2^ Interstitial nephritis.

**Table 4:** Number (n) of main and secondary pathological alterations. Positive findings (positive) versus no findings (negative). Diagnoses are based on necropsy of 64 rabbits reared in pens with the ground floor consisting of slatted plastic floor panels (Slats, n = 30) or pens with the ground floor consisting of concrete littered with straw litter (Straw, n = 34).

Affected Organs/Pathological Findings		Ground Floor Type		Total	Sig ^1^
		Slats	Straw		
Rhinitis	n negative	26	27	53	0.520
	n positive	4	7	11	
Diseases of the lower respiratory tract ^2^	n negative	16	15	31	0.617
	n positive	14	19	33	
Otitis	n negative	28	29	57	0.433
	n positive	2	5	7	
Conjunctivitis	n negative	28	32	60	1.000
	n positive	2	2	4	
Diseases of the heart ^3^	n negative	22	30	52	0.199
	n positive	8	4	12	
Nephritis	n negative	28	32	60	1.000
	n positive	2	2	4	
Stomach ulcers	n negative	29	31	60	0.616
	n positive	1	3	4	
Enteritis	n negative	8	10	18	1.000
	n positive	22	24	46	
Intestinal coccidiosis	n negative	7	6	13	0.757
	n positive	23	28	51	
Hepatic coccidiosis	n negative	30	33	63	1.000
	n positive	0	1	1	
Necrotising hepatitis	n negative	29	32	61	1.000
	n positive	1	2	3	
Emaciation	n negative	2	1	3	0.596
	n positive	28	33	61	

Note: ^1^ *p* values of Fisher’s exact tests; ^2^ Tracheitis, bronchitis, pleuritis, pneumonia; ^3^ Inflammation of myo- or pericard, myocard degeneration.

**Table 5:** Causes of loss attributed to body systems and affected organs/pathological findings across the three trials.

	% Affected within Trial			Chi-Square Test	
	Trial 1	Trial 2	Trial 3	χ^2^	*p*
**Causes of loss attributed to body systems**					
Digestive tract	43.75% (↓)	60.0%	83.33% (↑)	5.72	0.06
Digestive and respiratory tract	56.25% ↑	40.0%	8.33% ↓	8.27	0.02
Miscellaneous pathology	0%	0%	8.33%	4.40	0.11
**Affected organs/pathological findings**					
Rhinitis	28.13% (↑)	5.0% (↓)	8.33%	5.44	0.07
Diseases of the lower respiratory tract	65.63% ↑	55.0%	8.33% ↓	11.61	< 0.01
Otitis	12.50%	5.0%	16.67%	1.21	0.55
Conjunctivitis	9.38%	5.0%	0%	1.39	0.50
Diseases of the heart	28.13% (↑)	15.0%	0% (↓)	4.80	0.09
Nephritis	9.38%	0%	8.33%	1.96	0.38
Stomach ulcers	0%↓	5.0%	25%↑	9.39	0.01
Enteritis	62.50%	90% (↑)	66.67%	4.80	0.09
Intestinal coccidiosis	68.75%↓	100% ↑	75.00%	7.63	0.02
Hepatic coccidiosis	3.13%	0%	0%	1.02	0.60
Necrotizing hepatitis	9.38%	0%	0%	3.15	0.21
Emaciation	100% ↑	85.0% ↓	100%	6.93	0.03

Arrows in brackets indicate trends, arrows without brackets indicate significant differences between trials.

## Supplementary Materials

The following are available online at https://www.mdpi.com/2076-2615/9/5/216/s1, Data S1: data_windschnurer.et.al.xlsx.
